# Sensitivity of the Molecular Test in Saliva for Detection of COVID-19 in Pediatric Patients With Concurrent Conditions

**DOI:** 10.3389/fped.2021.642781

**Published:** 2021-04-12

**Authors:** Guzmán-Ortiz Ana Laura, Nevárez-Ramírez Abraham Josué, López-Martínez Briceida, Parra-Ortega Israel, Angeles-Floriano Tania, Martínez-Rodríguez Nancy, Jamaica-Balderas Lourdes, De la Rosa-Zamboni Daniela, Ortega-Riosvelasco Fernando, Jaramillo-Esparza Carlos Mauricio, Bonilla-Pellegrini Sergio René, Reyna-Trinidad Irineo, Márquez-González Horacio, Medina-Contreras Oscar, Quezada Héctor

**Affiliations:** ^1^Laboratorio de Investigación en Inmunología y Proteómica, Hospital Infantil de México Federico Gómez, Mexico City, Mexico; ^2^Laboratorio Clínico, Hospital Infantil de México Federico Gómez, Mexico City, Mexico; ^3^Unidad de Investigación Epidemiológica en Endocrinología y Nutrición, Hospital Infantil de México Federico Gómez, Mexico City, Mexico; ^4^Servicio de Neumología, Hospital Infantil de México Federico Gómez, Mexico City, Mexico; ^5^Departamento de Epidemiología Hospitalaria, Hospital Infantil de México Federico Gómez, Mexico City, Mexico; ^6^Departamento de Infectología, Hospital Infantil de México Federico Gómez, Mexico City, Mexico; ^7^Departamento de Enfermería, Hospital Infantil de México Federico Gómez, Mexico City, Mexico; ^8^Departamento de Investigación Clínica, Hospital Infantil de México Federico Gómez, Mexico City, Mexico

**Keywords:** adolescents, children, COVID-19, molecular diagnostics, saliva, SARS-CoV-2

## Abstract

**Background:** The reference standard for the molecular diagnostic testing for COVID-19 is the use of nasopharyngeal or combined nasopharyngeal and oropharyngeal (NP/OP) swabs. Saliva has been proposed as a minimally invasive specimen whose collection reduces the risks for health care personnel.

**Objective:** To assess the suitability of saliva for COVID-19 diagnosis as a replacement of the reference standard NP/OP swab in the setting of a tertiary care pediatric unit.

**Study design:** A paired study based in the prospective cohort design in patients suspected of having COVID-19.

**Methods:** RT-PCR was used to detect SARS-CoV-2 in paired samples of saliva and NP/OP swab collected from May through August 2020 from 156 pediatric participants, of whom 128 has at least one comorbidity and 91 showed clinical symptoms related to SARS-CoV-2 infection. Additionally, we studied a group of 326 members of the hospital staff, of whom 271 had symptoms related to SARS-CoV-2 infection.

**Results:** In the group of pediatric participants the sensitivity of the diagnostic test in saliva was 82.3% (95% CI 56.6–96.2) and the specificity 95.6% (95% CI 90.8–98.4). The prevalence of COVID-19 was 10.9% (17/156). In 6 of the 23 participants who tested positive for SARS-CoV-2 in at least one specimen type, the virus was detected in saliva but not in NP/OP swab, while in 3 participants the NP/OP swab was positive and saliva negative. In the group of adults, the sensitivity of the test in saliva was 77.8% (95% CI 67.2–86.3) and prevalence 24.8% (81/326). Discordant results between the two types of specimens showed a significant association with low viral load in the pharynx of adults but not of pediatric participants.

**Interpretation:** In the context of a pediatric tertiary care hospital, the sensibility of the test in saliva is not high enough to replace the use of NP/OP swab for COVID-19 diagnosis. Neither NP/OP swab nor saliva could detect all the participants infected with SARS-CoV-2.

## Introduction

Pediatric patients infected with the severe acute respiratory syndrome coronavirus 2 (SARS-CoV-2) who also have concurrent conditions are at high-risk to develop severe forms of the infection (1–3). Thus, in tertiary care pediatric hospitals, efficient programs for detection of COVD-19 in staff and patients are necessary to prevent the spread of the infection.

Currently, SARS-CoV-2 detection is based on real-time reverse transcription PCR (RT-PCR) amplification of viral genes from nasopharyngeal (NP), combined NP and oropharyngeal (OP), or nasal and oral swabs. This requires sampling by a trained health care worker who is exposed to aerosols from patients. Additionally, discomfort of the procedure may make some children uncooperative to this test. Saliva has been explored as alternative specimen for SARS-CoV-2 detection; it is minimally invasive and can be obtained by patients themselves.

Several studies have focused on the use of saliva to diagnose COVID-19 in adults, but the knowledge of the sensitivity of the test in saliva of children with concurrent conditions is scarce. Medication and immunosuppression of these patients may influence the viral load in the oral cavity.

Some studies have reported highly concordant results between upper respiratory tract swabs and saliva using posterior oropharyngeal saliva, coughed out saliva or sputum enriched saliva in adults (4–7). However, production of such specimens may be difficult for children. The use of early morning saliva collected just after waking, before eating, drinking or tooth brushing has also been explored (4–6, 8, 9), but in a hospitalization context, this requirement would prevent sampling at reception or before emergency procedures. Reported concordance rates between upper respiratory tract swabs and saliva have been highly variable (53–100%) (4–20). Currently there is no consensus on the use of different saliva sampling techniques, detection kits, RNA extraction methods, and the optimal time window for saliva collection after symptoms onset (21). If oral saliva could report the presence of the virus with high sensitivity when collected at any hour, it would be convenient in a pediatric hospitalization context.

The objective of this study was to assess the suitability of saliva for COVID-19 diagnosis as a replacement of the reference standard NP/OP swab in the setting of a tertiary care pediatric unit. To this end, the sensitivity and specificity of the SARS-CoV-2 detection test in saliva were estimated in children and adolescents, the majority of whom had concurrent conditions. In parallel, a group of adults, members of the hospital staff, was studied to compare our experimental approach with previous studies and to get a general picture of the diagnostic performance of saliva in those who interact in a health care setting. Given the importance of prevention of nosocomial infections, we hypothesized that the RT-PCR-based diagnostic test in saliva would show a minimum level of sensibility of 95% when the results of the NP/OP swab were used as reference standard in both, patients and staff.

## Materials and Methods

### Study Design

The prospective cohort design in suspected patients approach (22) was used to conduct a paired study in which the role of the test in saliva was replacement of NP/OP swab (23) for COVID-19 diagnosis.

This study followed the STARD 2015 recommendations for reporting diagnostic accuracy studies (24).

### Participants

**Figure 1:**
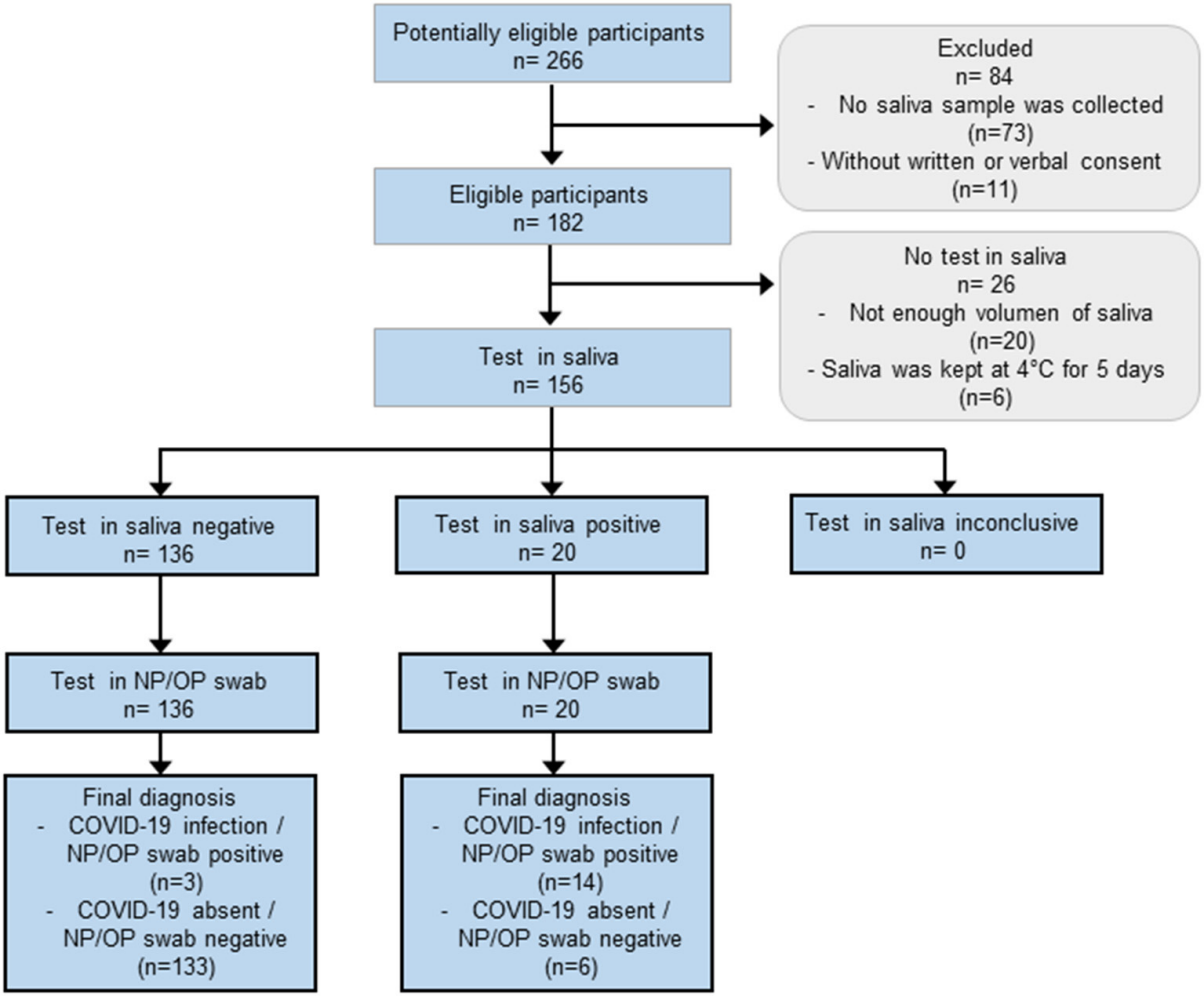
Flow of pediatric patients whose results were used to calculate the sensitivity and specificity of the test in saliva. This group was comprised of children and adolescents who attended to the hospital with clinical symptoms related to SARS-CoV-2 infection, hospitalized patients who showed respiratory symptoms while recovering from a disease other than COVID-19, and non-probable COVID-19 patients who attended to the hospital for routine clinical analyses before a programmed surgery. The characteristics of these participants are shown in Tables 1, 2.

**Figure 2:**
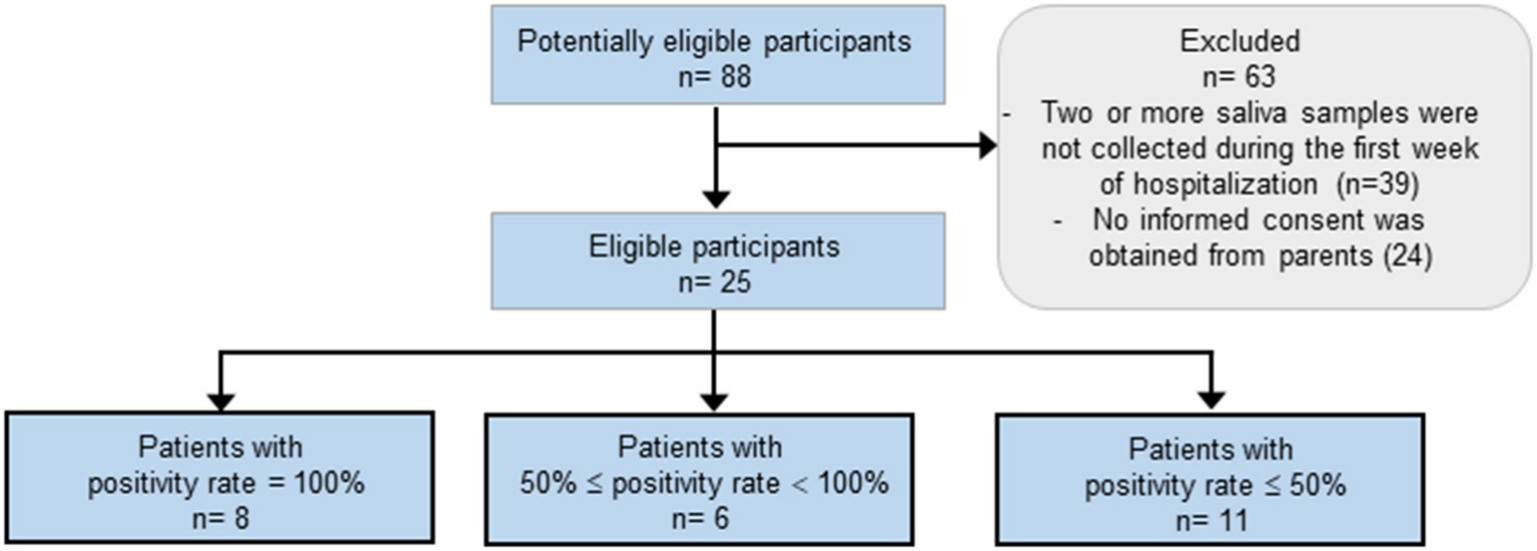
Flow of hospitalized pediatric patients with confirmed COVID-19 whose results were used to calculate the positivity rate during the first week of stay. The characteristics of these participants are shown in Table 3.

**Figure 3:**
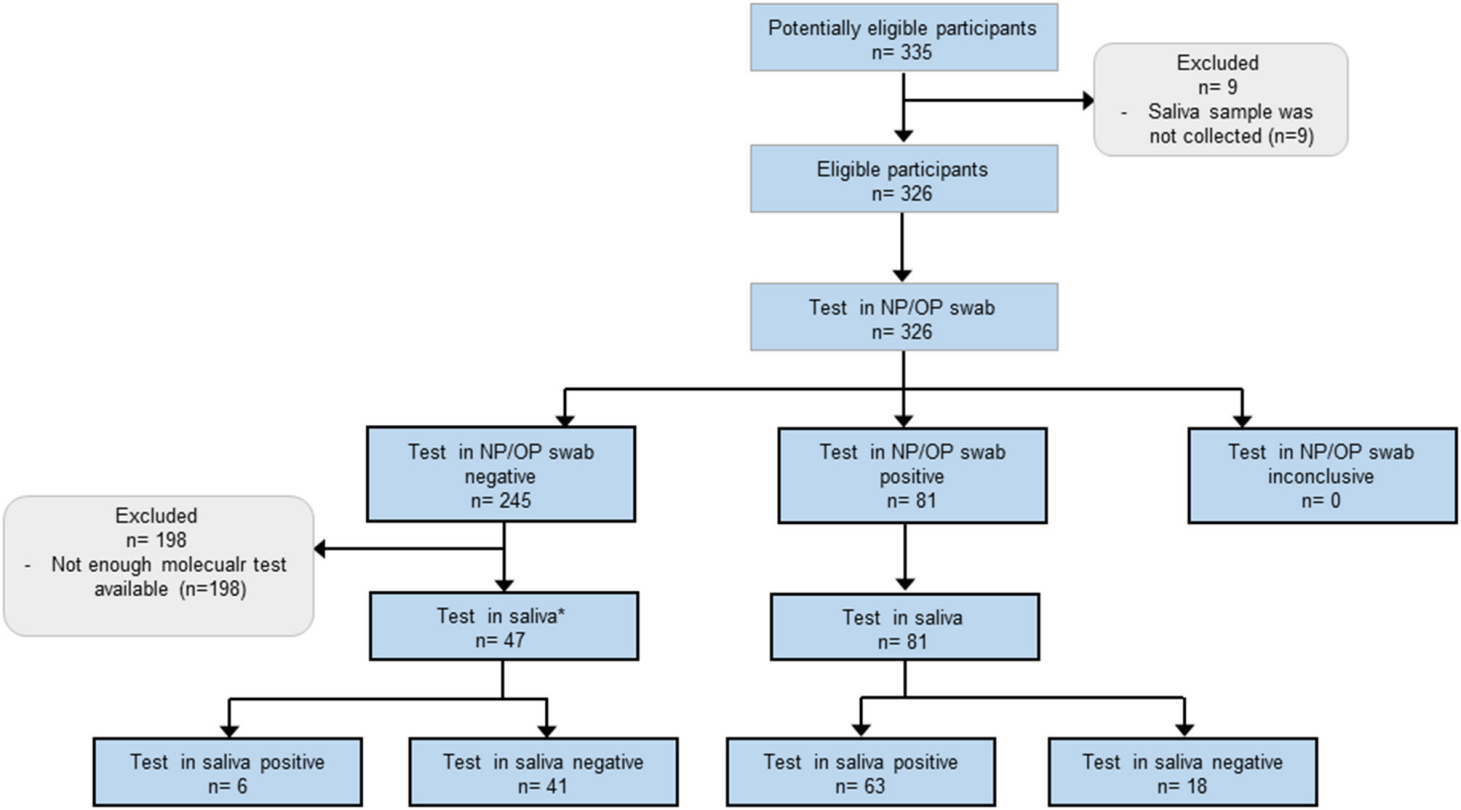
Flow of adult participants members of the hospital staff. *Due to shortage of molecular test, only 47 saliva samples were selected from the 245 participants who tested negative in the NP/OP swab. Selected samples were those from participants with higher possibilities to be infected with SARS-CoV-2: 30 participants who showed symptoms associated with COVID-19, and 17 participants in which the Ct values of the NP/OP swab analysis were just above 40, this value was the threshold for positivity. The characteristics of these participants are shown in Tables 4, 5.

Participants were patients or members of the hospital staff of the Hospital Infantil de México Federico Gómez, which is a tertiary care unit and a COVID-19 pediatric reference hospital in Mexico City. Three groups of participants were included in this study: (i) pediatric participants who were COVID-19 non-confirmed or non-suspected patients (Figure 1), results of this group were used for estimation of sensitivity and specificity of the test in saliva; (ii) pediatric participants confirmed positive for SARS-CoV-2 who were recovering at a COVID-19-specific area (Figure 2), results of this group were used to follow the course of the infection by collecting saliva samples during the first week of stay; and (iii) adult participants members of the hospital staff from all departments (Figure 3), results of this group were used for calculation of the sensitivity of the test in saliva from adults.

The eligibility criteria for pediatric participants were: at least 5 and no more than 18 years old, granted signed informed consent from the parents and verbal consent from participants, for suspected cases, the clinical criterion was the presence of at least one symptom related to SARS-CoV-2 infection. The exclusion criteria were declined to participate and not provide enough saliva sample.

The characteristics of the group of children and adolescents whose results were used for estimation of sensitivity and specificity are shown in Table 1. This group comprised 156 ambulatory and hospitalized participants, note that the reason of hospitalization in this group was a disease other than COVID-19. Potentially eligible participants were identified on the basis of their attendance to either the emergency room, or to a designated consulting room for sampling as part of their routine preoperative laboratory tests. In the case of hospitalized patients, eligible participants were identified on the basis of the presence of symptoms related to SARS-CoV-2 infection. Matched NP/OP swab and saliva sample pairs were collected from: (i) 54 non-probable COVID-19 patients who had a programmed surgery, (ii) 68 ambulatory participants with clinical symptoms related to SARS-CoV-2 infection, and (iii) 34 hospitalized patients showing symptoms related to SARS-CoV-2 infection at any hospital ward. In this way, a consecutive sample of pediatric participants was formed during the first COVID-19 wave in Mexico City, which began in early April and extended until late September 2020. Samples were collected between May 4th and August 28th. In this group of participants clinical information and reference standard results were not available to the performers or readers of the test in saliva, nor the results of saliva were available to performers or readers of the test in NP/OP swab.

**Table 1:** Clinical characteristics of the pediatric patients whose results were used to calculate sensitivity and specificity of the test in saliva.

**Characteristic**	**All** **(*n* = 156)**	**NP/OP swab positive** **(*n* = 17)**	**NP/OP swab negative** **(*n* = 139)**
**Age (y)**			
Distribution	11 (7–14)	11 (8–15)	11 (7–14)
5–11	83 (53.2)	9 (53.0)	74 (53.2)
12–18	73 (46.8)	8 (47.0)	65 (46.8)
**Sex**			
Male	78 (50)	10 (58.8)	68 (48.9)
Female	78 (50)	7 (41.2)	71 (51.1)
Weight (Kg)	34.5 (21.7–48.5)	37.7 (29.1–51.0)	33 (21.7–48.5)
**Vital signs**^**a**^			
Temp (°C)	36.6 (36.1–37.5)	37.4 (36.3–38.1)	36.6 (36.1–37.3)
RF	23.5 (20–27)	24 (22–28)	23 (20–27)
CF	118 (98–138)	125 (98–144)	118 (98–136)
SpO_2_ (%)	95.5 (94–97.5)	95 (91–98)	96 (94–97)
Contact COVID-19^b^	9 (5.8)	2 (11.7)	7 (5.0)
**Signs and symptoms**			
Asymptomatic	65 (41.6)	1 (5.9)	64 (46)^*^^c^
Number of symptoms	1 (0–2)	2 (1–4)	1 (0–2)^*^^d^
Sore throat	16 (10.2)	5 (29.4)	11 (7.9)^*^^e^
Cough	16 (10.2)	4 (23.5)	12 (8.6)
Fever	55 (35.2)	11 (64.7)	44 (31.6)
Headache	21 (13.5)	3 (17.6)	18(12.9)
Diarrhea	10 (6.4)	1 (5.9)	9 (6.5)
Muscle pain	8 (5.1)	2 (11.8)	6 (4.3)
Fatigue/Weakness	14 (9.0)	3 (17.6)	11 (7.9)
Rhinorrhea	10 (6.4)	2 (11.8)	8 (5.7)
Vomiting	21 (13.5)	2 (11.8)	19 (13.7)
Abdominal pain	29 (18.6)	3 (17.6)	26 (18.7)
Breathing difficulty	14 (9.0)	3 (17.6)	11 (7.9)
**Concurrent conditions**			
None	28 (17.9)	1 (5.9)	27 (19.4)
1	91 (58.4)	11 (64.7)	80 (57.6)
>1	37 (23.7)	5 (29.4)	32 (23)
Obesity	7 (4.5)	4 (23.5)	3 (2.1)^*^^f^
Cancer	45 (28.8)	6 (35.3)	39 (28.1)
Allergy/asthma	3 (1.9)	1 (5.9)	2 (1.4)
Chronic kidney disease	18 (11.5)	3 (17.6)	15 (10.8)
Chronic liver disease	4 (2.6)	0	4 (2.9)
Heart disease	8 (5.1)	1 (5.9)	7 (5.0)
Neurological disorders	8 (5.1)	0	8 (5.7)
Anemia	14 (9.0)	3 (17.6)	11 (7.9)
Autoimmune	15 (9.6)	1 (5.9)	14(10.1)
Diabetes	4 (2.6)	1 (5.9)	3 (2.1)
Surgery	7 (4.5)	0	7 (5.0)

Data are shown as median (25–75 percentiles) or n (%). RF, respiratory frequency; CF, cardiac frequency; SpO_2_, oxygen saturation.

a Vital signs were determined at admission for ambulatory participants and, at the day of sampling for hospitalized patients.

b contact COVID-19 means that participants had contact with a person infected with SARS-CoV-2, please see the Materials and Methods-Participants section for a definition of contact.

* Statistically significant (p < 0.05) NP/OP swab positive vs. negative.

c p = 0.001 (OR = 7.827; 95% CI 1.159–52.857; p = 0.001),

d p = 0.002 (OR = 1.416; 95% CI 1.090–1.839; p = 0.009),

e p = 0.044 (OR = 4.848, 95% CI 1.444–16.28; p = 0.017),

f *p = 0.045 (OR = 5.075; 95% CI 1.079–23.87; p = 0.040)*.

Additional eligibility criteria for the group of 25 hospitalized children and adolescents confirmed positive for SARS-CoV-2 who were recovering at a COVID-19-specific area during their first week of stay (Figure 2, **Table 3**), included a positive result of a RT-PCT test from NP/OP swab at day 1 of hospitalization, and at least two saliva samples collected during the first week of stay.

Eligibility criteria for adult participants were: to be members of the hospital staff, the presence of at least one symptom related to SARS-CoV-2 infection, or to have had contact with a person who tested positive for COVID-19, and granted informed consent. Exclusion criteria were declined to participate and not provide enough saliva sample. In this group, potentially eligible participants were identified on the basis of their attendance to a designated consulting room for epidemiological surveillance for workers. In this way, a consecutive sample of 326 participants was formed. Their characteristics are shown in **Table 4**. Samples were collected between May 4th and August 28th 2020. In this group of participants, the results of the test in saliva were not independent of the results of NP/OP swab because due to shortage of molecular test, not all saliva samples were tested (Figure 3), the 128 selected saliva samples were: 81 from participants who tested positive in the NP/OP swab, 30 from participants who showed symptoms associated with COVID-19 but their NP/OP swab tested negative, and 17 from participants with negative results in the NP/OP swab but in which the Ct values were just above 40, this value was the threshold for positivity.

In this work, those who had contact with a person infected with SARS-CoV-2, were those who met at least one of the following criteria: (a) proximity within 1.5 meters for at least 15 min to a confirmed case, while both the case and the contact were not continuously wearing mouth, nose and eye protection, (b) physical contact with a case without immediate hand hygiene, (c) contact with respiratory secretions, feces and vomit without immediate hand hygiene or use of gloves, and (d) being in a room where an aerosol-generating procedures were performed on a case while not wearing an N-95 mask and eye goggles. These criteria had to be met during the period of maximum contagiousness, i.e., from 48 h before the case's symptom onset and until 14 days afterwards.

None of the participants were severe or critical patients of COVID-19 at the time of sampling. The protocol was approved by the ethical committee of the Hospital Infantil de México Federico Gómez (HIM-2020-026). Written informed consent was obtained from adult participants and parents of children and adolescents, verbal assent was obtained from pediatric participants.

### Specimen Collection and SARS-CoV-2 Detection

NP/OP swab and saliva matched pairs for each participant were collected in the same day. The NP/OP swabs were collected by trained healthcare workers in the same tube containing 2 ml of 1X Hanks' balanced salt solution without phenol red (Thermo Fisher Scientific, Waltham, MA, USA). Immediately after the swab procedure, saliva was collected by the participants after being instructed to gently spit 5 times into a sterile 50 ml centrifuge tube, they were not instructed to cough out or try to enrich their samples with sputum. No clinical interventions were made between collection of the two samples. Specimens were kept at room temperature up to 4 h and then processed for viral RNA extraction or, if collected at late evening, kept at 4°C overnight and processed the following morning. Samples were taken at any hour of the day.

Viral RNA extractions were made from 140 μl of sample with the QIAamp Viral RNA mini kit (QIAGEN, Hilden, Germany) and eluted in 60 μl. Saliva specimens with high viscosity were diluted with an equivalent volume of 1X Hanks' balanced salt solution without phenol red (Thermo Fisher Scientific, Waltham, MA, USA). Detection of SARS-CoV-2 was done by RT-PCR with 5 μl of RNA template using the GeneFinder COVID-19 Plus RealAmp kit (ELITechGroup, Puteaux, France) (25) which amplifies the viral RdRP, E and N genes as well as the human RP gene as internal control. Following manufacturer's instructions. Briefly, a master mix was prepared containing all components of the reaction including enzymes, nucleotides, probes and flourophores; 15 microliters of this mix were added per well in a 96-well-plate and 5 microliters of extracted RNA were then added. The PCR program comprised two segments. Segment one: 1 cycle at 50°C for 20 min and 1 cycle at 95°C for 5 min. Segment two: 45 cycles of 95°C for 15 s and 58°C for 60 s. Valid results were those in which the internal control gene was amplified with a Ct ≤ 35. A sample was considered positive if at least one of the RdRp, N or E genes were amplified with a Ct ≤ 40. Negative samples were those in which no amplification of any viral genes was observed, and the internal control was amplified with a Ct ≤ 35. No indeterminate results were observed in this study, but these were defined as those tests in which the internal control gene were not amplified with a Ct ≤ 35. At the beginning of the study, and because of irregular supply of molecular tests, some NP/OP swabs samples were analyzed with the Daan kit (Da An Gene Co., Ltd. of Sun Yat-sen University, Guangzhou, China) (26) which amplifies the viral ORF1ab and N genes, and RNase P as internal control. In these cases (4 children and 17 adults) the criteria for positivity were amplification of at least one viral gene and the internal control with Ct ≤ 40, and for negative results, amplification of only the internal control with a Ct ≤ 40. Cut-offs for positivity were pre-specified by the manufacturers of the commercial kits.

The viral copy number was estimated extrapolating the threshold cycle for gene N (Ct_geneN_) on a standard curve obtained by 10-fold serial dilutions of a plasmid containing the complete nucleocapsid gene from SARS-CoV-2, the resulting equation was:

(1) $$\begin{array}{l} \frac{\text{Viral copies}}{\text{ml saliva}}\;=10^{\frac{\text{CtgeneN }-\;38.09}{-\;3.2251}}\;*\;85.7 \end{array}$$

This standard curve was made by the researchers for this study using the above mentioned GeneFinder COVID-19 Plus RealAmp kit.

### Statistical Analyses

The values of sensitivity and specificity were estimated using the results of the NP/OP swap as reference standard (19–21) as described by Linnet and coworkers (22). The overall percent agreement was calculated following FDA recommendations (27). Comparison of the detection performance of paired saliva and swabs samples was done using McNemar's test. As alternative measures of agreement between results of saliva and NP/OP swabs, the overall percent agreement and κ statistics were estimated (27, 28). Results of NP/OP swabs were used as reference standard for calculation of sensitivity and specificity (19–21). To analyze the potential association of clinical variables with the concordance rate between saliva and NP/OP swab, we used χ^2^ test to compare proportions and calculate risk. The logistic regression models were constructed including one variable at a time, and final models included biological variables and variables with statistical significance. Confounding bias was accepted when changes in estimated odds ratios (ORs) were equal or larger than 10%. When a principal effect model was reached, effect modification was also tested, and interaction terms were constructed between the positivity in NP/OP swabs and variables shown in Tables 1, **4**. The terms were included in the model when the significance of the *p*-value was larger or equal to 0.20. The likelihood ratio test was performed for each multiple logistic model. To compare numerical variables, the Mann–Whitney *U*-test was used or the Wilcoxon test for matched samples, data are presented as median and percentiles 25 and 75. Comparison of categorical variables was done with χ^2^ or Fisher's exact test and data are shown as absolute frequencies and proportion. Statistical significance was set at *p* ≤ 0.05. Statistical analyses were performed using GraphPad PRISM v8 or Stata v14.0.

If one result of a matched pair was lost or unavailable because not enough volume of saliva was collected or any other reason, the whole pair was removed from the study.

The sample size was calculated as described by Flahault and coworkers (29). The expected sensitivity was 0.85, prevalence 0.1 and the minimum acceptable lower confidence limit was 0.5. This resulted in a *n* = 180 (18 cases and 162 controls). The expected sensitivity value was set based on the average reported agreement rate between results from saliva and upper respiratory tract swabs which is 84.4% (4–7, 10–18). We estimated a prevalence of 10 % based on the positivity rate observed in patients attending to the emergency room at the beginning of the study. We did not reach the intended sample size mainly because saliva sample was not collected and lack of written or verbal consent (Figure 1).

## Results

### The Estimated Sensitivity of the Test in Saliva of Pediatric Patients Was Below the Hypothesized Value

The group of potentially eligible pediatric participants comprised 266 children and adolescents, however, 84 were excluded because saliva samples were not collected, or because written or verbal consent were not obtained. Other 26 participants were excluded due to the low volume of saliva collected or because samples were stored for too long at 4°C (Figure 1). Thus, for determination of the sensitivity and specificity of the test in saliva of children and adolescents, we compared results from 156 NP/OP swab and saliva matched pairs from 122 ambulatory and 34 hospitalized participants. Note that the reason for hospitalization in these patients was a disease other than COVID-19. SARS-CoV-2 was detected in 14/23 participants in both NP/OP swab and saliva samples, 3/23 tested positive in NP/OP swab only, and 6/23 tested positive in saliva only (Table 2). Using the NP/OP swab results as reference standard, the sensitivity and specificity of the test in saliva were 82.3% (95% CI 56.6–96.2) and 95.6% (95% CI 90.8–98.4), respectively.

**Table 2:** Concordance of the SARS-CoV-2 detection rate in saliva and NP/OP swab in children and adolescents.

	**Positive in NP/OP swab**	**Negative in NP/OP swab**	**Total**
Positive in saliva	14	6	20
Negative in saliva	3	133	136
Total	17	139	156

Baseline demographic and clinical characteristics of participants are shown in Table 1. The median (interquartile) age was 11 (7–14), 50% were male and 128 (82%) had at least one concurrent condition being the most frequent cancer, chronic kidney disease, autoimmune disorders, and anemia. Ninety-one participants (58.3%), showed at least one symptom associated with COVID-19. The most frequent symptoms were fever, abdominal pain, headache and vomiting. All the participants with SARS-CoV-2 infection were patients with mild or moderate disease and none of them evolved to severe respiratory disease. The median (interquartile) time between onset of symptoms and sampling was 2 (1–5) days.

The prevalence of COVID-19 was 10.9% (95% CI 6.4–16.8) and 12.8% (95% CI 8.0–19.1) based on results of NP/OP swab or saliva, respectively.

**Figure 4:**
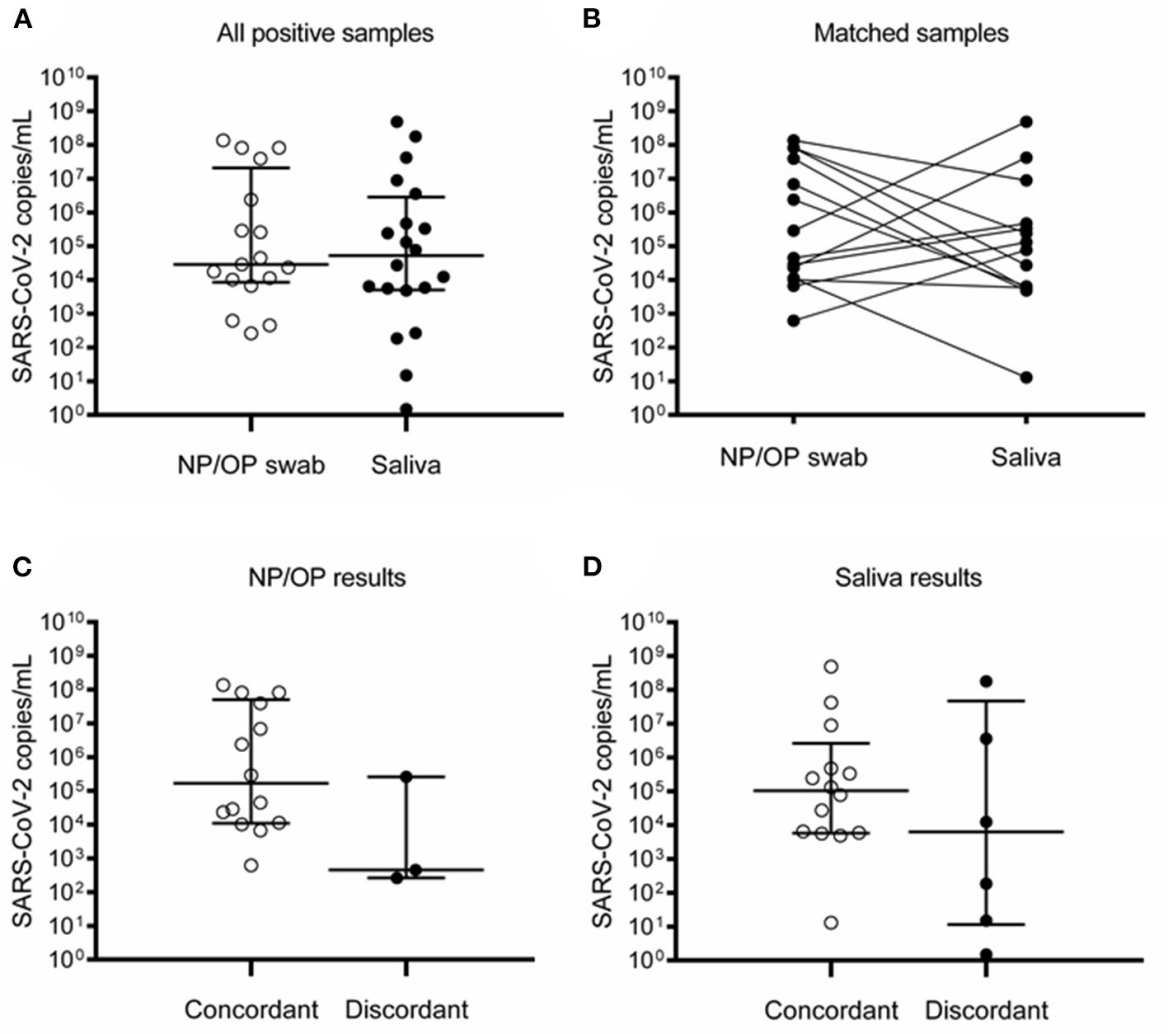
Detection of SARS-CoV-2 in NP/OP swabs and saliva of children and adolescents. **(A)** All positive samples in NP/OP swab (*n* = 17) and saliva (*n* = 20) (*p* = 0.6565), bars represent median and 25–75 percentiles. **(B)** Matched samples (*n* = 14) (*p* = 0.5093). **(C)** Viral loads of concordant (*n* = 14) and discordant (*n* = 3) NP/OP swab samples (*p* = 0.0924). **(D)** Viral loads of concordant (*n* = 14) and discordant (*n* = 6) saliva samples (*p* = 0.2542). Data in **(A,C,D)** were compared with Mann–Whitney test. Data in **(B)** were compared with Wilcoxon test.

McNemar's test indicated that SARS-CoV-2 detection rates were similar in both specimens (*p* = 0.5078). As alternative statistical measures of agreement, calculation of the overall percent agreement resulted in 94.2% (95% CI 89.3–97.3), and a kappa coefficient of 0.724 (95% CI 0.639–0.809). Viral loads did not show a significant difference between NP/OP swab and saliva (Figures 4A,B). Due to the low number of samples and high variability, results did not show enough statistical power to compare the viral loads between concordant and discordant samples (Figures 4C,D).

Comparison of clinical variables between participants who tested positive vs. negative in NP/OP swab, only revealed significant differences in presence and number of symptoms as well as the occurrence of sore throat and obesity (Table 1).

### Use of Saliva to Follow the Infection of Pediatric Patients With Concurrent Conditions

To evaluate the potential of saliva as specimen to follow up the course of the infection, we collected saliva samples from confirmed patients along their first week of hospitalization. The flow of these participants is shown in Figure 2. From 88 potentially eligible hospitalized pediatric patients, 63 were excluded because <2 saliva samples were collected during the first week of stay, or because no informed consent was obtained from the parents. All participants tested positive in a NP/OP swab at day 1. Although the number of saliva samples collected was different among participants (it varied from 2 to 5), some participants clearly showed higher positivity rates than others (Figure 2, Table 3). Comparison of the characteristics of those with positivity rates higher or equal than 50% vs. those with <50% revealed a significant difference only in the viral load of the NP/OP swab collected at day one of hospitalization (p=0.0128). All participants with viral loads >8.72 × 10^6^ (Ct_geneN_ = 21.9) in the swab, showed positivity rates ≥50%; whereas only 35.2% of participants with viral loads ≤ 3.02 × 10^4^ (Ct_geneN_ = 33.1) in the swab, showed positivity rates higher or equal than 50% in saliva (Table 3).

**Table 3:** Positivity rates in saliva of children and adolescents with confirmed COVID-19 during their first week of hospitalization.

**Child**	**Age (y)**	**Sex**	**Concurrent condition**	**Ct _**geneN**_ of NP/OP swab at day 1**	**Viral load in NP/OP swab at day 1 (copies/ml)**	**First week of hospitalization (days)**							**Positivity rate**	**Total days of hospitalization**
						**1**	**2**	**3**	**4**	**5**	**6**	**7**		
						**Result in saliva**								
1	5	F	Epilepsy	15.2	1.07 × 10^9^	–	–	1	–	1	–	–	100	14
2	16	F	ALL, obesity	16.2	4.99 × 10^8^	–	–	1	1	–	–	–	100	17
3	5	F	HIV, herpes zoster, Ramsay Hunt syndrome, oral candidiasis, malnutrition	18.2	1.25 × 10^8^	1	–	1	–	1	–	–	100	12
4	12	M	Chondroblastic osteosarcoma	21.0	1.70 × 10^7^	–	1	1	–	–	1	–	100	9
5	16	M	Renal transplantation	30.2	2.27 × 10^4^	–	1	–	1	1	–	–	100	5
6	15	M	ALL, obesity	31.2	1.13 × 10^4^	–	1	1	–	–	–	–	100	14
7	16	M	Renal insufficiency	31.4	1.01 × 10^4^	–	1	–	–	1	–	–	100	6
8	14	F	None	36.5	2.66 × 10^2^	–	–	1	–	1	–	–	100	6
9	5	F	Anorectal malformation, unilateral renal agenesis	18.8	8.20 × 10^7^	1	–	–	1	1	0	1	80	12
10	6	F	Acute nephrotic syndrome	15.9	6.50 × 10^8^	–	1	0	–	–	1	–	67	14
11	17	F	ALL, seizure crisis	17.3	2.39 × 10^8^	–	–	0	1	–	–	–	50	8
12	7	M	ALL	21.9	8.72 × 10^6^	–	–	1	–	0	0	1	50	11
13	12	F	Obesity, acute kidney failure	37.0	1.89 × 10^2^	–	0	–	–	–	1	–	50	6
14	17	F	Psychiatric disorder, asthma	37.1	1.72 × 10^2^	–	–	0	–	1	–	–	50	6
15	15	M	Anemia, pneumonia with pleural effusion, Hepatosplenomegaly	37.2	4.52 × 10^2^	0	1	–	–	0	0	–	25	15
16	11	M	Aplastic anemia, sepsis	30.5	1.93 × 10^4^	–	–	0	–	–	0	–	0	10
17	5	M	Appendicitis	33.1	3.02 × 10^4^	–	–	0	0	–	–	–	0	13
18	13	M	ALL	33.2	2.81 × 10^4^	–	–	0	–	–	0	–	0	9
19	5	M	ALL	33.8	1.82 × 10^4^	–	–	–	0	–	0	0	0	15
20	11	F	Ewing's sarcoma	34.6	1.03 × 10^4^	–	0	–	0	–	–	–	0	7
21	17	F	Synovial sarcoma	35.2	6.79 × 10^2^	–	–	0	0	–	–	0	0	7
22	9	F	ALL	36.1	3.44 × 10^2^	–	0	0	–	–	0	–	0	7
23	13	F	Renal insufficiency	36.2	3.33 × 10^2^	–	0	0	–	–	0	–	0	6
24	6	M	Appendicitis	36.7	2.31 × 10^2^	–	0	–	–	0	–	–	0	8
25	16	F	Appendicitis	37.79	1.06 × 10^2^	0	–	0	–	–	–	–	0	4

*ALL, acute lymphoblastic leukemia; HIV, human immunodeficiency virus. Positivity rate represents the percentage of positive samples among all the collected samples for each patient during the first week of hospitalization. 1, positive result; 0, negative result; –, no sample was collected*.

### The Sensitivity of the Test in Saliva Was Similar in Hospital Staff and Pediatric Patients

To evaluate the potential of saliva as specimen for epidemiological surveillance in hospital staff, we collected 326 NP/OP swab and saliva matched pairs. The group of potentially eligible adult participants was formed by 335 adults (Figure 3). However, 9 were excluded because saliva sample was not collected. The remaining 326 NP/OP swab samples were analyzed but, due to the shortage of molecular tests, not all the saliva samples were tested. This resulted in exclusion of 198 saliva samples (Figure 3). From the 326 NP/OP swab, 81 resulted positive, their corresponding saliva specimens were then tested and 63 resulted positive (estimated sensitivity of 77.8%, 95% CI 67.2–86.3). In participants who tested negative in NP/OP swab, 30 were ordered to stay at home for 14 days in isolation because at the time of sampling, they showed symptoms associated with COVID-19, their corresponding saliva samples were analyzed and one of them tested positive (Figure 3). The Ct values of other 17 samples with negative results in NP/OP swab were just above 40 which was the threshold for positivity, the corresponding saliva samples were tested and 5 of them were positive (Figure 3).

**Figure 5:**
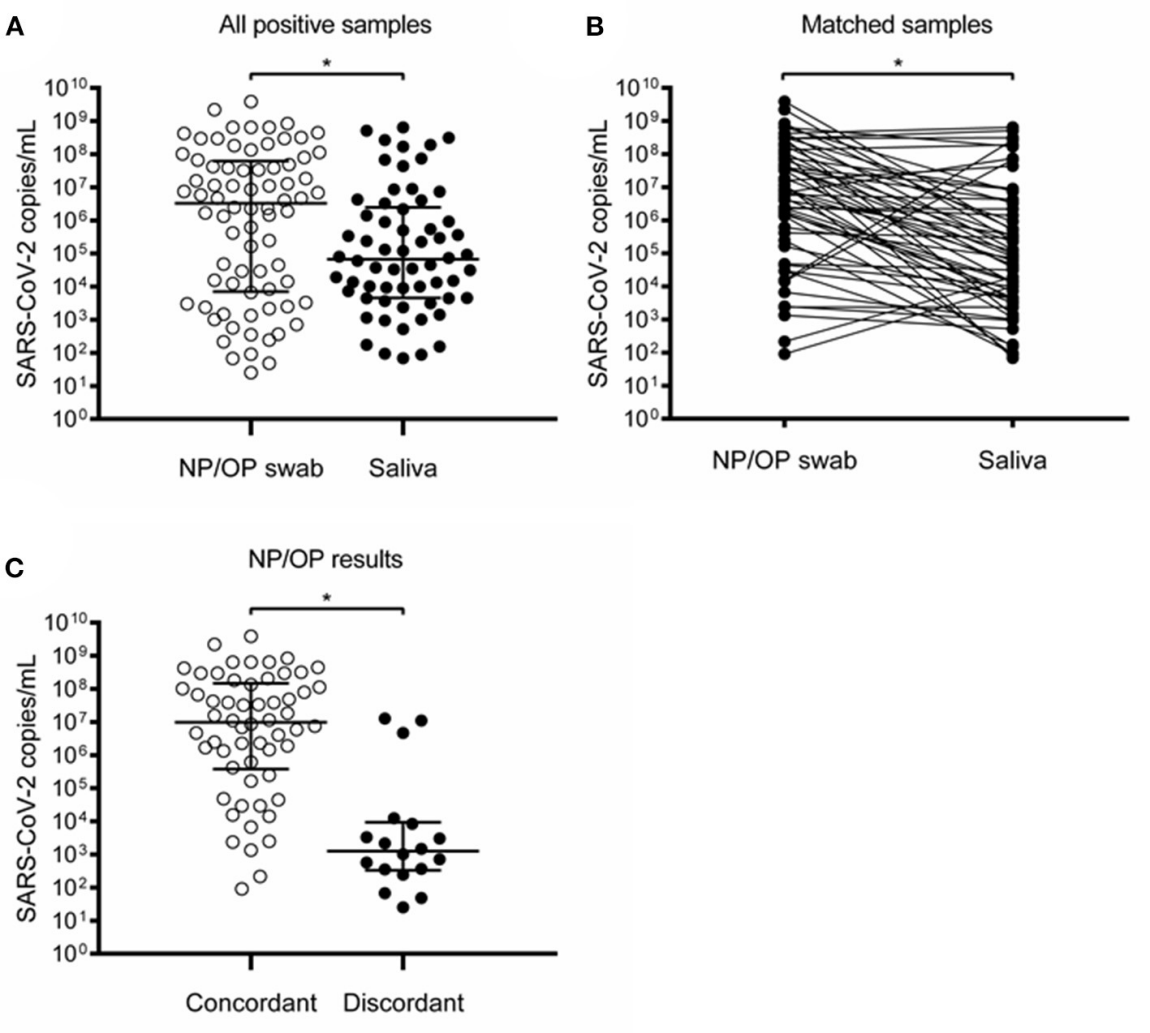
Detection of SARS-CoV-2 in NP/OP swabs and saliva of adults. **(A)** All positive samples in NP/OP swab (*n* = 81) and saliva (*n* = 63) (*p* = 0.0161), bars represent median and 25–75 percentiles. **(B)** Matched samples (*n* = 63) (*p* = 0.0001). **(C)** Viral loads of concordant (*n* = 63) and discordant (*n* = 18) NP/OP swab samples (*p* < 0.0001). Data in **(A,C)** were compared with Mann–Whitney test. Data in **(B)** were compared with Wilcoxon test. *Statistically significant (*p* < 0.05).

The characteristics of the group adults are shown in Table 4. The median (interquartile) age was 37 (30–47), 111 (34%) were men, and 250 (76.6%) had at least one concurrent condition, the most frequents were overweight (36%), obesity (32%) and hypertension (9%). As expected, the presence and number of symptoms, headache, fever, anosmia, and ageusia, as well as the occurrence of at least one comorbidity, showed significant association with positive result in the NP/OP swab (Table 4). Of the 81 participants who tested positive for SARS-CoV-2 in NP/OP swab, 74 (91%) showed symptoms associated with COVID-19 and the median (interquartile) time between symptoms onset and sampling was 3 (1–5) days. All the participants with SARS-CoV-2 infection showed mild or moderate illness and none of them evolved to severe respiratory disease. The prevalence of COVID-19 was 24.8% (95% CI 20.2–29.9) based on results of NP/OP swab. Viral load in NP/OP swab resulted significantly higher than that in saliva (*p* = 0.0161; Figures 5A,B, Table 5). Moreover, viral load in NP/OP swab of the 63 concordant samples was significantly higher than that of the 18 discordant samples (*p* < 0.0001; Figure 5C).

**Table 4:** Clinical characteristics of the adult participants included in this study.

**Characteristic**	**All** **(*n* = 326)**	**NP/OP swab positive** **(*n* = 81)**	**NP/OP swab negative** **(*n* = 245)**
Age (y)	37 (30–47)	38 (30–46)	37 (30–47)
**Sex**			
Men	111 (34)	33 (40.7)	78 (31.8)
Women	215 (66)	48 (59.3)	167 (68.2)
Contact COVID-19	196 (60.1)	47 (58)	149 (60.8)
**Signs and symptoms**			
Asymptomatic	55 (16.9)	7 (8.6)	48 (19.6)*^a^
Number of symptoms	4 (2–6)	5 (3–6)	4 (1–6)*^b^
Headache	193 (59.2)	56 (69.1)	137 (55.9)*^c^
General malaise	143 (43.8)	36 (44.4)	107 (43.7)
Sore throat	132 (40.5)	33 (40.7)	99 (40.4)
Cough	109 (33.4)	34 (42)	75 (30.6)
Rhinorrhea	83 (25.4)	26 (32)	57 (23.2)
Fever	73 (22.4)	33 (40.7)	40 (16.3)*^d^
Diarrhea	51 (15.6)	8 (9.9)	43 (17.6)
Thoracic pain	50 (15.3)	14 (17.3)	36 (14.7)
Breathing difficulty	40 (12.3)	14 (17.3)	26 (10.6)
Abdominal pain	27 (8.3)	2 (2.5)	25 (10.2)
Anosmia	19 (5.8)	13 (16)	6 (2.4)*^e^
Vomiting	15 (4.6)	4 (4.9)	11 (4.5)
Ageusia	14 (4.3)	9 (11.1)	5 (2.0)*^f^
**Concurrent conditions**			
None	76 (23.3)	17 (21)	59 (24.1)
≥1	250 (76.7)	64 (79.0)	186 (75.9)*^g^
Overweight	118 (36.2)	30 (37)	88 (35.9)
Obesity	104 (31.9)	28 (34.6)	76 (31)
Hypertension	29 (8.9)	8 (9.9)	21 (8.6)
Diabetes	12 (3.7)	3 (3.7)	9 (3.7)
Asthma/COPD	15 (4.6)	5 (6.1)	11 (4.5)
Heart disease	4 (1.2)	0	4 (1.6)
Immune disease	4 (1.2)	1 (1.2)	3 (1.2)
Smoke	23 (7.1)	4 (4.9)	19 (7.7)

Data are shown as median (25–75 percentiles) or n (%). *Statistically significant (p < 0.05).

a p = 0.023 (OR = 0.388; 95% CI 0.168–0.896; p = 0.027),

b p = 0.003 (OR = 1.140; 95% CI 1.043–1.246; p = 0.004),

c p = 0.036 (OR = 1.765; 95% CI 1.034–3.014; p = 0.037),

d p < 0.001 (OR = 3.523; 95% CI 2.016–6.155; p < 0.001),

e p < 0.001 (OR = 7.615 95% CI 2.789–20.786; p < 0.001),

f p < 0.001 (OR = 6; 95% CI 1.948–18.471; p = 0.002),

g *p = 0.022 (OR = 1.467; 95% CI 0.820–2.623; p = 0.196)*.

**Table 5:** Viral loads and concordance rates in variables with significant differences among adult participants who tested positive in the NP/OP swab.

**Participants**	**Viral load in NP/OP swab**	**Viral load in saliva**	**Number of positive saliva samples (concordance rate)**
All (*n* = 81)	3.2 × 10^6^ (7.9 × 10^3^-5.3 × 10^7^)^^*^a^	6.7 × 10^4^ (5.2 × 10^3^-2 × 10^6^) ^^*^a^	63 (78%)
Men (*n* = 33)	5.9 × 10^6^ (6.1 × 10^5^-4.8 × 10^7^)	1.0 × 10^5^ (1.3 × 10^4^-3.4 × 10^7^)	30 (91%)^^*^b^
Women (*n* = 48)	2.6 × 10^5^ (2.8 × 10^3^-3.9 × 10^7^)	3.3 × 10^4^ (2.3 × 10^3^-4.9 × 10^5^)	33 (69%)^^*^b^

Data are shown as median (25–75 percentiles) or n (%).

* Statistically significant (p < 0.05).

a viral load in NP/OP swab from all participants was significantly higher than viral load in saliva (p = 0.016),

b *The concordance rate between saliva and NP/OP swab was significantly lower in women than in men (p = 0.018; OR 0.22, 95% CI 0.05–0.88, p = 0.019)*.

The concordance rate between positive results in NP/OP swab and saliva was significantly lower in women than in men (68.8 vs. 90.9%, *p* = 0.018; OR 0.22, 95% CI 0.05–0.88, *p* = 0.019; Table 5). Association of viral loads, concordance rates, and other clinical or work-related variables did not reveal significant differences.

None of the participants, either pediatric or adults, experienced adverse events from performing the test in saliva or in NP/OP swab.

## Discussion

The use of oral saliva collected by the patients themselves at any hour of the day would be convenient for children in the context of a tertiary care unit as an alternative specimen for COVID-19 diagnosis. This approach resulted in an estimated sensitivity of 82.3%, when the result of NP/OP swab was taken as reference standard in a group of children and adolescents, the majority of whom had comorbidities, and of 77.8% in members of the hospital staff. These values were below the set value of our hypothesis (95%). Our interpretation of these results is that, although the use of oral saliva could reduce the risk of infection of health care workers and discomfort of the patients, it does not show enough sensitivity to replace the NP/OP swab for COVID-19 diagnosis in the context of a tertiary care hospital.

Currently, the reference standard for SARS-CoV-2 detection is the use of respiratory tract specimens, mainly NP swab (19–21); and it is likely to remain so in tertiary care units because of the high variability of reported sensitivity values in saliva which ranges from 53 to 100% (4–7, 10–20), the values observed in our study are within this range.

It has been reported that the RT-PCR-based detection of SARS-CoV-2 from NP swabs and other upper respiratory tract specimens for COVID-19 diagnostics show sensitivity values that range between 71 and 97% (30, 31). The use of systematic reviews and meta-analysis has led to the conclusion that the sensitivities of the COVID-19 diagnostic test in saliva and NP swab are not substantially different, which makes the use of saliva an attractive option for SARS-CoV-2 detection in a community setting (19, 20). In these meta-analysis, definition of the reference standard included positive results in either NP swab or saliva. Considering that NP swab is an imperfect standard (19), a positive result in saliva but negative in NP/OP swab could be interpreted as presence of COVID-19. These types of results from matched pairs are frequently found (6, 8, 9, 11, 13, 16, 18, 21, 32, 33). In the group of children and adolescents whose results were used to estimate the sensitivity and specificity of the test in saliva (Tables 1, 2), 6 participants showed positive results in saliva but negative results in the corresponding NP/OP swabs. One of them was re-tested collecting a NP/OP swab 2 days after the matched pair was collected and the result of the second NP/OP swab was positive. Other three, were diagnosed with pneumonia, other showed headache, fever and sore throat, the sixth participant was asymptomatic. Further research is necessary to establish if a positive result in saliva but negative in NP/OP swab should be considered as an evidence of SARS-CoV-2 infection and to determine the actual contribution of saliva to reduce the number of missed cases among pediatric patients with suspected COVID-19 treated in tertiary care units.

The use of alternative measures of agreement in the group of children and adolescents whose results were used to estimate the sensitivity and specificity of the test in saliva (Tables 1, 2, Figure 1), resulted in an overall percent agreement of 94.2% but in a moderate agreement as judged by the kappa coefficient (0.724), whereas the McNemar's test indicated that detection rates were similar in saliva and NP/OP swab (*p* = 0.5078). The use of an imperfect reference standard (19, 20) and the low number of samples contribute to the statistical uncertainty of our results.

A clear difference between pediatric and adult participants was that saliva showed significantly lower viral loads than NP/OP swabs in adults but not in children and adolescents (Figures 4B, 5B). Although the number of participants with positive matched samples was different between these two groups, this observation may reflect differences in the dynamics of viral shedding in the oral cavity associated with age, comorbidities, or medication.

One of the factors that contribute to discordant results between NP/OP swabs and saliva seems to be low viral load. In our group of adults, viral loads in NP/OP swabs were significantly higher in concordant than in discordant pairs (Figure 5C). However, in the group of children and adolescents who were ambulatory patients or hospitalized for a disease other than COVID-19 (Tables 1, 2, Figure 1), statistical power was not enough to test hypotheses between pairs with concordant vs. discordant results due to the low number of samples (Figures 4C,D).

In the group of hospitalized children and adolescents (Table 3), participants with high viral load in NP/OP swabs collected at day 1 of hospitalization showed higher positivity rates in saliva during the first week of stay. However, this group of patients is small, and few samples were collected. This prevents precise estimations of the potential of saliva to follow up the course of the infection and precise associations among variables. Still, the differences in positivity rates shown in Table 3 suggest that the sensitivity of the test in saliva as a function of time along the course on the infection, is influenced by the viral load during the early days of infection. A sharp decrease in viral load of saliva in children during the first 10 days of the infection has been reported (34), probably this effect contributed to the predominance of negative results observed in some participants (Table 3). A negative result in saliva of hospitalized children, may mean low infectivity because infection of cells in culture has been observed almost exclusively for specimens with viral loads ≥1 × 10^6^ (35–37), and 85.3% of negative results in Table 3 were below this value.

Besides viral load, we sought significant associations between other clinical variables and the concordance rate between NP/OP swab and saliva. In the group of children and adolescents however, we found no associations, probably because of the low number of infected participants, or the heterogeneity of concurrent conditions.

Of note, none of the infected pediatric participants either in the group of patients with suspected COVID-19 (*n* = 23, Table 2) or in the group of confirmed patients (*n* = 25, Table 3) evolved to a severe form of COVID-19 even though 95.8% (46/48) had at least one concurrent condition and many of them were taking immunosuppressant drugs.

In the group of adults statistically significant associations were found between the SARS-CoV-2 infection and symptoms commonly found in this condition like headache, fever, anosmia or ageusia (Table 4). In the group of pediatric participants (Table 1), such symptoms were not significantly associated with a positive result in NP/OP swab. It may be possible that the heterogeneity of medication and concurrent conditions in children and adolescents prevented such statistical significance. In the group of adults, a lower concordance rate was observed in women than in men (Table 5) which probably resulted from a trend of women to show lower viral loads in saliva (3.3 × 10^4^ and 1.0 × 10^5^ for women and men, respectively) (Table 5), although this difference was not significant (*p* = 0.064).

One of the strengths of this study is that the inclusion of asymptomatic non-probable cases, suspected cases, and hospital staff, allowed a general picture of the diagnostic performance of oral saliva in a pediatric tertiary care unit. The prevalence of COVID-19 in the group of children and adolescents was around 10%, and that of workers was 24.8%. The high prevalence in the group of adults is the result of the selection procedure, samples were collected only from members of the hospital staff who attended to a designated consulting room for epidemiological surveillance for workers who showed symptoms or who had contact with a confirmed case.

One of the limitations of our study was the low number of patients with confirmed COVID-19 in children and adolescents and the high heterogeneity of concurrent conditions. The low sample size led to wide 95% CI for estimates of sensitivity and specificity. Generalization of such estimates must be done in the context of pediatric participants with underlying conditions who are medicated and treated at a tertiary care unit. This study did not include COVID-19 patients treated at the intensive care unit; thus, a potential source of bias is the inclusion of children with mild and moderate illness. Pediatric participants were 5 years old or older, our results are not applicable to younger children. As sensitivity of the test in saliva depends on the anatomical origin of the sample (20, 21), our results are biased to the use of saliva collected with the spitting technique. Additionally, in our study viral RNA was manually extracted with commercial kits, this may prevent generalization to other settings because in many laboratories RNA extraction is made with automated platforms. Further studies with defined subpopulations and more participants are necessary to determine the extent to which the sensitivity of the test in saliva is influenced by clinical or community-related variables like prevalence, socioeconomic status, or other social determinants of health.

In the case of adults, the main limitation was the low number of saliva samples analyzed from participants whose NP/OP swab tested negative. Generalization of our estimated sensitivity value is limited to adults with mild or moderate illness in a hospital setting. Inclusion of employees from all areas of the Hospital, prevented bias toward subgroups of workers, like those who interact with patients for example.

## Data Availability Statement

The raw data supporting the conclusions of this article will be made available by the authors, without undue reservation.

## Ethics Statement

The studies involving human participants were reviewed and approved by Ethical committee, Hospital Infantil de México Federico Gómez, Mexico City, Mexico (HIM-2020-026). Written informed consent to participate in this study was provided by the participants' legal guardian/next of kin.

## Author Contributions

G-OA, N-RA, and QH transported samples, extracted RNA, registered, and analyzed clinical information. L-MB, P-OI, A-FT, and M-CO made RT-PCR analysis and registered clinical information. M-RN, DD, and O-RF performed statistical analyses. J-BL, J-EC, B-PS, and R-TI collected samples. G-OA and M-GH collected informed consents. QH wrote the manuscript and designed the protocol. The full original study protocol can be obtained from the corresponding author. All authors approved the final manuscript.

## Conflict of Interest

The authors declare that the research was conducted in the absence of any commercial or financial relationships that could be construed as a potential conflict of interest.
